# The impact of the Ponseti treatment method on parents and caregivers of children with clubfoot: a comparison of two urban populations in Europe and Africa

**DOI:** 10.1007/s11832-016-0719-7

**Published:** 2016-02-22

**Authors:** Francesc Malagelada, Sadia Mayet, Greg Firth, Manoj Ramachandran

**Affiliations:** Paediatric Orthopaedics, Department of Orthopaedic and Trauma Surgery, Royal London Hospital, Barts Health NHS Trust, Whitechapel Road, London, E1 1BZ UK; Paediatric Orthopaedics, Department of Orthopaedic Surgery, Chris Hani Baragwanath Academic Hospital, Johannesburg, South Africa

**Keywords:** Ponseti, Clubfoot, Congenital talipes equinovarus, Stress, Coping, Social support

## Abstract

**Purpose:**

With the Ponseti treatment method established as the gold standard, children with clubfeet face a prolonged treatment regime that might impact on their families. We aimed to determine how Ponseti treatment influences the lives of parents and caregivers and what coping strategies they use. Secondarily, we aimed to identify any potential differences between two urban referral centres for clubfoot.

**Methods:**

A total of 115 parents of children affected with idiopathic clubfoot were recruited and included in two groups: one from the United Kingdom (UK) and the other from South Africa (SA). The participants completed the following three instruments: the Impact on Family Scale (IOFS), the Multidimensional Scale of Perceived Social Support (MSPSS), and the Brief COPE.

**Results:**

During the bracing phase, the IOFS showed a trend towards lower scores when compared to the casting phase for both cohorts (*p* = 0.247 and *p* = 0.434, respectively). The SA population scored higher than the UK in the MSPSS in both casting (*p* = 0.002) and bracing phases (*p* = 0.004) and used coping strategies at a significantly higher level when compared to the UK population (*p* < 0.05) in both treatment phases.

**Conclusion:**

This is the first study to show that Ponseti treatment for clubfoot causes an impact on family function. In SA, perceived social support is higher and coping strategies are used more often than in the UK to deal with the stressful circumstances of treatment.

## Introduction

Congenital talipes equinovarus (CTEV) or clubfoot is the most common musculoskeletal deformity at birth, with a reported incidence of 1.2 per 1000 live births [1]. Various treatment options exist for CTEV but the current accepted gold standard is the Ponseti method, with open surgery reserved for those feet that cannot be completely corrected [2–5].

Parents of children diagnosed with CTEV face increased stress during pregnancy and/or at birth [6]. In addition, they have to learn to accept the deformity in the neonatal period, to attend weekly visits to the hospital for several weeks after birth for casting and to cope daily with an intensive bracing regime until the child is at least 3 or 4 years old.

It is known that chronic conditions in children, such as asthma, heart and renal disease, cause increased levels of anxiety, depression and stress in parents [7–9]. Only one study reports preliminary findings of a negative impact of the diagnosis of CTEV on the mother’s psychological well-being [6]. It is important to evaluate the parent’s perspective in order to minimize the impact of the condition and to implement appropriate interventions that help optimize family function.

There are no published studies that evaluate the effect of the currently accepted gold standard treatment of CTEV on parents and caregivers. We conducted a double-centre study of parents with children diagnosed with idiopathic CTEV to determine the impact on the family of the serial casting and bracing stages of the Ponseti treatment regime [10]. We also investigated any potential differences in impact and illness perception between two different communities: the high-income city of London, United Kingdom, versus the low-income city of Johannesburg, South Africa [11].

## Materials and methods

### Patients

We recruited the parents or caregivers of 115 children (one for each child) diagnosed with idiopathic clubfoot who were undergoing Ponseti treatment in the form of bracing (standard Denis Browne boots and bar). Participants were recruited from two units experienced in treating clubfoot with the Ponseti method: The Royal London Hospital (RLH) in Whitechapel, London, UK (50 cases) and The Chris Hani Baragwanath Academic Hospital (CHBAH), Soweto, Johannesburg, South Africa (65 cases).

The criteria for inclusion in the study were (1) parents or caregivers of children with a clinical diagnosis of idiopathic CTEV (unilateral or bilateral), (2) who were able to fill in the questionnaires, (3) who were able to give parental informed consent to partake in the study, (4) whose affected children were aged under 5.5 years and (5) were undergoing Ponseti treatment at the time of the study (the foot abduction boots and bar phase). Exclusion criteria were (1) children with underlying neuromuscular disorders or associated conditions, e.g. arthrogryposis, (2) children with relapse of clubfoot requiring further surgery (apart from percutaneous heel cord tenotomy), (3) parents or caregivers unable to consent to partaking in the study.

The study was approved by the local audit and clinical effectiveness committees of both the RLH and CHBAH, and written consent to use the data was obtained from the parents or guardians of the children. Patients who fulfilled the inclusion criteria were selected and their parents or caregivers offered the opportunity to participate in the study. Once consent was obtained, parents or caregivers were given a booklet with the questionnaires and demographic information to be completed.

Three questionnaires were administered: the Impact on Family Scale (IOFS), the Multidimensional Scale of Perceived Social Support (MSPSS), and the Brief COPE. Each parent was asked to answer the three questionnaires at two different time points: (1) serial casting phase and (2) bracing phase (foot abduction boots and bar). The data for the casting phase of treatment was collected retrospectively when parents or caregivers filled in the questionnaires during the bracing phase of treatment. Questionnaires were administered in English for both populations in their validated form during clinic appointments. In cases of illiteracy or difficulty in completing questionnaires, a member of staff assisted those parents in order to permit inclusion in the study.

The results of the questionnaires were analysed statistically using the unpaired Student *t* test for comparison between populations and paired Student *t* test for comparison of treatment stages within the same population. A *p* value of <0.05 was considered to be of statistical significance.

### Impact outcome measures

#### Impact on Family Scale (IOFS)

This scale is a 24-item quality of life instrument that evaluates the impact that a child’s illness has on family function. The revised version includes 24 items with responses to each of these on a four-point scale (from strongly agree to strongly disagree). An overall score ranges from 15 to 60. Internal consistency (Cronbach’s) for overall impact and for each domain ranges from 0.60 to 0.88 [12].

#### The Multidimensional Scale of Perceived Social Support (MSPSS)

This scale includes 12 seven-point items assessing the current perceived social support received either from family friends or the significant other. The sum of these scores is the global score of social support that ranges from 12 to 84 [13].

#### The Brief COPE

This is a questionnaire that assesses the range of coping strategies in stressful situations. The treatment involving either casting or boots and bars was referred as the stressful event. It is formed by 28 items grouped into 14 subscales. Response options range from 1 (I haven’t been doing this at all) to 4 (I’ve been doing this a lot). There is no “overall score” on this measure and instead each subscale has a score ranging from 2 to 8 showing which coping strategies have been used against the stressful situation [14].

### Clinical outcome measures

The Pirani score at diagnosis and at the latest follow-up were assessed and the number of casts applied and the need for Achilles tenotomy at any stage of the treatment were documented [15]. The children’s past medical history and any complications as a consequence of Ponseti treatment were taken into consideration.

## Results

### Epidemiology and treatment

The mean age of the children at the time of recruitment was 24.8 months (range 4–63), with the majority being male (68 %). Of all the affected children, 40 % were first-borns and a majority of these had no significant medical history (94 %) or any family history of clubfoot (87 %). Seven children were also diagnosed individually with asthma, exotropia, eczema, developmental dysplasia of the hip, bilateral curly toes, hypermobility and complicated birth. 55 % had unilateral clubfoot whereas the remaining 45 % were bilateral (Table 1).

**Table 1:** Demographics

Characteristic	UK (*n* = 50)	SA (*n* = 65)	Total (*n* = 115)
Age, months (range)	24.8 (4−59)	26.2 (4–63)	24.8 (4–63)
Male sex, no. (%)	30 (60)	48 (74)	78 (68)
Side, no. (%)			
Right	9 (18)	25 (38)	34 (30)
Left	15 (30)	14 (32)	29 (25)
Bilateral	26 (52)	26 (40)	52 (45)
Carer working status, no. (%)			
Both working	15 (30)	13 (20)	28 (24)
One working	28 (56)	46 (71)	74 (64)
None working	7 (14)	6 (9)	13 (12)
First-born, no. (%)	23 (46)	23 (35)	46 (40)
Siblings, mean no. (range)	1.3 (0–5)	1.0 (0–4)	1.1 (0–5)
Family history of CTEV, no. (%)^a^	5 (10)	10 (15)	15 (13)
Associated comorbidities, no. (%)	7 (14)	0 (0)	7 (6)

*UK* United Kingdom, *SA* South Africa, *M:F* male:female, *CTEV* congenital talipes equinovarus

^a^Including direct family: parents, grandparents or siblings

Ponseti treatment was initiated for all infants with a mean of 7.7 casts, and 74 % required percutaneous heel cord tenotomy. The Pirani score at diagnosis was on average 4.59, which improved to 0.14 at the latest follow-up, and 76 % of the cases experienced no difficulties or complications during treatment (Table 2).

**Table 2:** Ponseti treatment method

Characteristic	UK (*n* = 50)	SA (*n* = 65)	Total (*n* = 115)
Number of casts (range)	6.49 (2–12)	8.7 (1–24)	7.7 (1–24)
TA tenotomy, no. (%)	27 (54)	58 (89)	85 (74)
Pirani score at diagnosis (range)^a^	4.73 (2–6)	4.50 (1–6)	4.59 (1–6)
Pirani score at latest follow-up (range)^a^	0.11 (0–1.5)	0.17 (0–2.5)	0.14 (0–2.5)
Difficulties during treatment, no. (%)	18 (36)	9 (14)	27 (24)
Intolerance/PS, no. (%)	6 (12)	0 (0)	6 (5)
Non-compliance, no. (%)	4 (8)	1 (2)	5 (4)
Recurrence, no. (%)	8 (16)	8 (12)	16 (14)

*UK* United Kingdom, *SA* South Africa, *PS* pressure sores, *TA* Tendo Achilles

^a^In bilateral cases the highest score was considered

Social data was compared between populations. For the UK a deprivation rank was used and for SA the household income (Table 3).

**Table 3:** Families’ deprivation/income

Percentile	UK, *n* (%)^a^	SA, *n* (%)^b^
25th	35 (70)	20 (32)
50th	11 (22)	28 (43)
75th	3 (6)	15 (23)
100th	1 (2)	2 (2)
*n*	50	65

Number and percentage of families within each percentile range for United Kingdom (*UK*) and South Africa (*SA*). Lower percentiles indicate higher deprivation or lower income

^a^Deprivation taken from the Indices of Deprivation 2010 of all neighbourhoods in England. Source: Office for National Statistics. Lower figures indicate higher deprivation [17]

^b^Household incomes in 2008 from the National Income Dynamic Survey (NIDS) [18]

### Questionnaires

The results of both IOFS and MSPSS questionnaires are shown in Table 4. Families showed similar levels of impact in both populations and for both treatment stages. South African families showed a higher level of perceived social support during both stages of treatment (*p* < 0.05).

**Table 4:** Results of questionnaires

Questionnaire	UK population	SA population	Total	*p* value A
IOFS casting (range)	29.0 (15–54)	29.6 (15–52)	29.3 (15–54)	0.746
IOFS bracing (range)	26.7 (15–53)	28.6 (15–57)	27.7 (15–57)	0.281
*p* value B	0.247	0.434	0.226	
MSPSS casting (range)	62.1 (21–84)	70.5 (41–84)	66.9 (21–84)	**0.002**
MSPSS bracing (range)	62.9 (22–84)	70.8 (42–91)	67.3 (22–91)	**0.004**
*p* value C	0.543	0.755	0.813	

Statistically significant p values are in bold (p < 0.05)

*UK* United Kingdom, *SA* South Africa, *IOFS* Impact on Family Scale, *MSPSS* Multidimensional Scale of Perceived Social Support

*p* values: *A* comparison of the means of UK versus SA population, *B* comparison of the means of IOFS during casting versus bracing periods, *C* comparison of the means of MSPSS during casting versus bracing periods

**Fig. 1:**
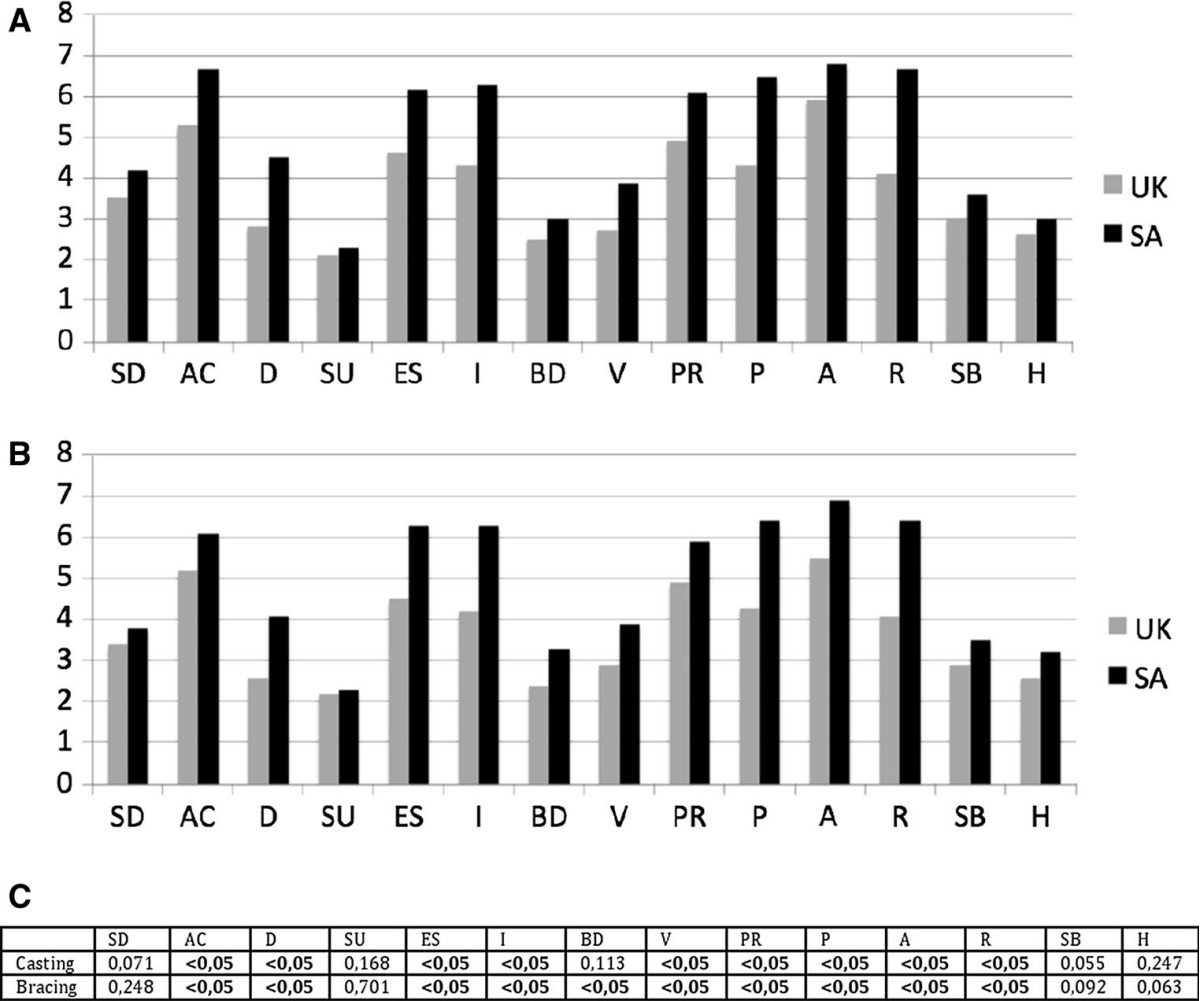
Results of the Brief COPE and level of coping strategies used during the casting phase (**a**) and the bracing phase (**b**) amongst the two populations. *UK* United Kingdom, *SA* South Africa, *SD* self distraction, *AC* active coping, *D* denial, *SU* substance use, *ES* emotional support, *I* instrumental, *BD* behavioural disengagement, *V* venting, *PR* positive reframing, *P* planning, *A* acceptance, *R* Religion, *SB* self blame, *H* humour. **c** Comparison of the strategies used between the two populations and statistical significance at each phase of treatment

Coping strategies used by parents according to the Brief COPE were analyzed and the results are shown in Fig. 1. Overall, the strategies most frequently used were from the Active Coping and Acceptance categories. The SA population demonstrated a higher use of coping strategies when compared to the UK for both the casting and the bracing phases. When comparing the two stages of treatment within each of the populations, there were no significant differences in the use of any of the categories.

## Discussion

The results of this study objectively demonstrated the impact of Ponseti treatment on parents and caregivers of children affected with clubfoot. Despite being very successful, relatively non-invasive and embraced by the medical community as the gold standard, medical professionals need to acknowledge that this treatment regime causes increased stress for the families.

For a similar impact on the family, South African parents perceive higher social support, as measured using the MSPSS, and employ significantly more coping strategies, according to Brief COPE, than their British peers. This may be due to cultural and social differences between the studied populations. Our cohorts from the two different countries were dissimilar in terms of ethnicity and so the effect of the condition and its treatment, and the ways that stress is handled, are likely to be expressed differently. The results of our study show that South Africans are more comfortable receiving support and that they tend to search more for external collaboration with and help from friends and family. They employed nine different coping strategies at a significantly higher level than the British cohort in both the casting and bracing phases. These included active coping, denial, emotional support, instrumental, venting, positive reframing, planning, acceptance and religion. In the remaining five coping categories of the Brief COPE, the British cohort still showed a lower level of use although this was not statistically significant. Although it is true that South African children underwent on average a higher number of casts (6.9 UK vs 8.7 SA), when ignoring the effect of the outliers the median values are very similar for both populations (6 UK vs 7 SA). Such a small difference of one extra cast is unlikely to produce an effect on perceived social support or impact in families. Our study contributes to the literature on the assessment of cultural differences in coping with chronic conditions and the effect that a child’s medical treatment has on different cultural backgrounds [16]. Most research in this field has been conducted in Western cultures and to our knowledge none has compared European and African populations.

Social data showed that a vast majority of the families in our cohort were below the 50th percentile for household income or deprivation (92 % in the UK and 75 % in SA). We suspect that the observed lower levels of income and higher deprivation may play a role in the level of stress and coping strategies used by families, although we cannot prove this in our study. Different variable units were used in our populations, the reason being that the Office for National Statistics in the UK provided data on deprivation but this was not available for SA. In the latter population we used household income instead [17, 18].

There are some limitations of this study. Firstly, the lack of a control group does not allow for comparison with a cohort of families with healthy babies. Secondly, all questionnaires were collected retrospectively and this could have led to recall bias. To minimize this effect, we only included patients who were close to the initial casting phase so that the parents could recall their experience more easily. Thirdly, we accept that there may be an underlying gender-related bias in answering the questions, as we did not specifically ask the gender of the person completing the questionnaires. Previous evidence indicates that mothers report more psychological stress than fathers [19, 20]. Nevertheless, with the numbers in the study, we expect that this would balance out between the two groups and clearly the person most engaged with the child’s care would likely attend the appointments and would therefore be the most appropriate person to answer the questions.

A recently published study by Coppola et al. [6] is the only paper to our knowledge that investigates how an orthopaedic physical malformation can interfere with the mother’s psychological well-being. Mothers were questioned in the first 3 months after giving birth to a child diagnosed with CTEV and compared to mothers of healthy full-term babies. Amongst others, the Brief COPE and MSPSS were used. They found that mothers in the CTEV group reported more stress-related and depressive symptoms in reaction to the birth of their child and found a protective role for social support. Moreover, they highlighted the importance of implementing protocols in the hospital unit directed to parents of babies with a congenital malformation. Our study focuses on the treatment of CTEV and its impact on families who have already been through the negative event of being diagnosed at birth. Our results show only a trend towards higher impact during the initial casting phase when compared to bracing. It is unclear whether this trend is due to the higher demand of the treatment itself or to the residual effect shown by Coppola et al. [6] after being diagnosed at birth. It is likely that during the first 3 months after birth, the impact of the diagnosis could be superimposed on the effect of the casting treatment. It is therefore during this first month of treatment that families have the highest need for medical and psychological support.

In chronic diseases in children, the literature supports the need for parents to have access to medical information and emotional support [8, 9, 21, 22]. On completion of this study, a support protocol was implemented in the UK unit for parents undergoing treatment for clubfoot. A website was updated with information on clubfoot treatment available to all parents. Close links with the charity Steps (http://www.steps-charity.org.uk) were developed in order to provide guidance and support for those parents in need, including a helpline and an online community of parents that aids to reassure families of children with clubfoot. In SA the local charity Steps (http://www.steps.org.za) provide two full-time parent support staff who speak to parents and help them to cope with the stress of having a child undergoing the Ponseti treatment. Similar strategies have proved effective in managing uncertainty in the context of clubfoot [23]. Finally, a virtual clinic led by a specialized physiotherapist was implemented in the UK centre which allows consultation with parents and the child from home using videoconferencing technology. This has received very positive initial feedback, as parents complained that the number of follow-up visits and journeys to the treating hospital were one of the most significant burdens of treatment. There are plans for a similar clinic to be set up in the South African centre.

Interventions that support and help parents during the Ponseti treatment method are vital in order to minimize the impact on families. In children with chronic conditions, parenting stress and marital satisfaction can be significantly affected in an adverse manner [24]. Possible future avenues of research could include new treatment methods that reduce the numbers of visits to hospital while maintaining the standards of care, and providing the parents with easily accessible support.

## Conclusion

This is the first study to assess the impact of an orthopaedic treatment in parents of affected children and to consider cultural differences between the two populations. The findings show that both stages of Ponseti treatment, serial casting and bracing, have a similar impact on families, although the initial casting stage demonstrated a trend towards higher impact. The populations studied showed significant differences in the perceived social support and the employment of coping strategies, with both being higher in SA than the UK. Implementation of protocols that support parents are recommended in an attempt to improve the well-being of families from different cultural and social backgrounds.
